# Prolonged Prothrombin Time due to Drug–Drug Interaction of Warfarin after the Change from Bosentan to Macitentan: A Case of Pharmacist Intervention in the Outpatient Clinic

**DOI:** 10.24546/0100492951

**Published:** 2025-02-03

**Authors:** TOMOKO KURIMURA, TOMOHIRO OMURA, KAZUHIRO YAMAMOTO, HIDEKAZU TANAKA, TAKESHI KIMURA, KOTARO ITOHARA, YUMI KITAHIRO, YASUSHI HABU, TOSHIYASU SAKANE, IKUKO YANO

**Affiliations:** 1 Department of Pharmacy, Kobe University Hospital, Kobe, Japan; 2 Department of Integrated Clinical and Basic Pharmaceutical Sciences, Faculty of Medicine, Dentistry and Pharmaceutical Sciences, Okayama University, Okayama, Japan; 3 Division of Cardiovascular Medicine, Department of Internal Medicine, Kobe University Graduate School of Medicine, Kobe, Japan; 4 Kobe Pharmaceutical University, Kobe, Japan

**Keywords:** Warfarin, Bosentan, PT-INR, Drug-drug interaction, Pharmacist intervention

## Abstract

A woman in her 70s who was taking warfarin 3.75 mg/day had a prothrombin time-international normalized ratio (PT-INR) within the therapeutic range. Her medication for pulmonary hypertension was changed from bosentan to macitentan. After 40 days, she developed respiratory distress, anorexia, and vomiting caused by common cold. When she visited the pharmaceutical outpatient clinic without reservation, the pharmacist suspected that bosentan discontinuation, which cancelled cytochrome P450 (CYP) 2C9 and CYP3A4 enzyme induction, and decreased vitamin K intake due to appetite loss had enhanced warfarin effect, causing PT-INR prolongation. The pharmacist requested the physician to examine the patient’s PT-INR. Results showed that her PT-INR was >7. Hence, she was urgently hospitalized. Warfarin and macitentan were discontinued, and the patient’s PT-INR decreased to 1.77 after the intravenous administration of vitamin K. Her appetite improved, and warfarin 2 mg/day was resumed. Additionally, when she had been administered macitentan, her hemoglobin levels decreased from 10.8 to 6.6 mg/dL. Therefore, the pharmacist and the physician during hospitalization planned to resume treatment with bosentan, but not with macitentan. The pharmacist proposed to increase the warfarin dose to 3.75 mg since the bosentan and warfarin interaction could lower PT-INR. Thereafter, the patient’s PT-INR was controlled within the therapeutic range, and her hemoglobin level was 8–9 mg/dL. The patient was discharged on day 17 of admission. Thus, pharmacist intervention plays a significant role in warfarin control with consideration of drug–drug interaction in patients receiving pulmonary hypertension treatment.

## INTRODUCTION

The pharmaceutical outpatient clinic at Kobe University Hospital accepts all patients who underwent medical examinations performed by a specific physician at a specific time slot in the department of cardiovascular internal medicine. At the pharmaceutical outpatient clinic, the pharmacist meets the patient prior to the medical examination to check for medication adherence, confirm previous prescriptions, conduct physical assessments, and provide consultation services. In addition to providing feedback to physicians based on details gathered from patient interviews, the pharmacist accompanies patients during medical examinations, requests additional laboratory tests, or proposes prescriptions to physicians as necessary from a pharmaceutical perspective [1, 2].

Warfarin interacts with several drugs. If warfarin is used in combination with other drugs that induce or inhibit the activity of cytochrome P450 (CYP) 2C9 or CYP3A4, which is the enzyme responsible for metabolizing warfarin, warfarin effects are attenuated or enhanced, respectively, affecting prothrombin time-international normalized ratio (PT-INR) [3]. Thus, one must be cautious when switching or changing concomitant medications with warfarin. In this case, the pharmacist at a pharmaceutical outpatient clinic found an excessively prolonged PT-INR due to a drug–drug interaction of warfarin associated with the change from bosentan to macitentan, which are pulmonary hypertension medications, and continued to provide pharmaceutical interventions through the prescription proposal during hospitalization, resulting in an improvement of the patient’s condition and subsequently led to discharge.

## CLINICAL CASE

The patient was a woman in her 70s. She regularly visited the outpatient clinic at the department of cardiovascular internal medicine, Kobe University Hospital, due to pulmonary vascular hypertension, chronic atrial fibrillation (CAF), aortic valve replacement (AVR), mitral valve replacement (MVR), and post-tricuspid valve annuloplasty. The oral medications administered when visiting the pharmaceutical outpatient clinic were as follows: warfarin, 3.75 mg/day; macitentan, 10 mg/day; furosemide, 40 mg/day; esomeprazole, 20 mg/day; magnesium oxide, 750 mg/day; calcium polystyrene sulfonate jelly, 15 g/day; sildenafil, 60 mg/day; levothyroxine sodium, 125 mg/day; carvedilol, 5 mg/day; amlodipine, 5 mg/day; and ferrous citrate sodium tablet, 50 mg/day.

The patient presented with respiratory distress, anorexia, and vomiting caused by common cold 1 week prior to her visit without reservation at the outpatient clinic of the department of cardiovascular internal medicine. At that time, she visited the pharmaceutical outpatient clinic before the medical examination, and the pharmacist conducted a patient interview after confirming the patient’s previous drug history and laboratory test values. The patient was taking warfarin at a dose of 3.75 mg/day for CAF and postoperative AVR/MVR. Her PT-INR was within the therapeutic range (1.5–3.0) for >6 months prior to this visit. A review of her medication history revealed that during a routine visit to the outpatient clinic 1 month prior, her pulmonary hypertension medication was changed from bosentan 250 mg/day to macitentan 10 mg/day. The reason for changing the medication was unclear because there was no detail about it in the electronic medical record. The pharmacist considered the possibility that the decrease in vitamin K intake due to anorexia for 1 week and the change from bosentan to macitentan may have resulted in prolongation of PT-INR, because bosentan discontinuation cancels CYP2C9 and CYP3A4 enzyme induction, and requested the physician to measure the patient’s PT-INR. Based on the laboratory examination result, the patient’s PT-INR was >7. Hence, she was urgently hospitalized to assess for bleeding status and to adjust the warfarin dose. In addition, her hemoglobin (Hb) level decreased to 6.6 mg/dL, which indicated anemia.

Treatment with warfarin and macitentan was discontinued after hospitalization. On day 1 of admission, the patient received 10 mg of intravenous vitamin K. On day 2, her PT-INR was 1.77, which was within the therapeutic range. After admission, the patient’s common cold symptoms and appetite improved. Therefore, warfarin therapy was resumed at a reduced dose of 2 mg/day. On day 5, the patient’s PT-INR was 2.06, which was also within the therapeutic range. On day 9, the physician planned to resume the patient’s pulmonary hypertension medication during hospitalization. Since the progression of anemia could be an adverse effect of macitentan, the pharmacist and physician during hospitalization had a discussion, and treatment with bosentan at a dose of 250 mg/day for pulmonary hypertension medication was resumed. Warfarin interacts with bosentan, and the patient’s PT-INR can decrease again if both warfarin and bosentan are administered. Hence, the pharmacist proposed to increase the dose of warfarin from 2 to 3.75 mg, which was the same dose of warfarin in the concomitant administration with bosentan before. The physician agreed with the proposal of the pharmacist. After increasing the dose of warfarin to 3.75 mg on day 9, the patient’s PT-INR was 1.73 and 2.20 on days 11 and 17 of admission, respectively. Then, the patient was discharged on day 17.

**Figure 1:**
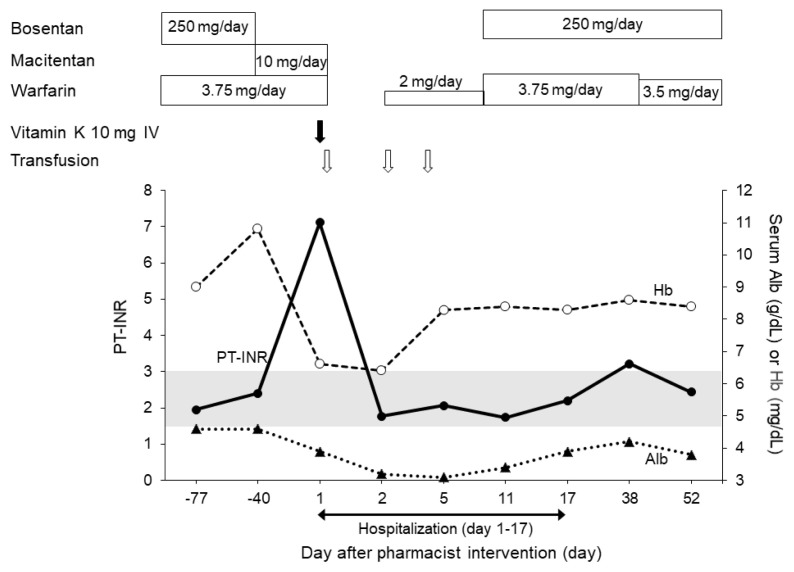
Warfarin dose, pulmonary hypertension medications, and laboratory test values. The patient received 10 mg of intravenous vitamin K on day 1 of admission, and transfusion therapy was performed on days 1, 2, and 4 of admission PT-INR, prothrombin time-international normalized ratio; Hb, hemoglobin; Alb, albumin. The shaded area shows that the PT-INR was within the therapeutic range (1.5–3.0).

For anemia, transfusion therapy was performed on days 1, 2, and 4 of admission. Upper endoscopy was performed, and bleeding was not found. The patient’s Hb level improved to 8.3 mg/dL on day 4 and remained at 8.3 mg/dL on the day of discharge. Figure 1 depicts the warfarin dose, pulmonary hypertension medication, and laboratory test values in this case.

## DISCUSSION

In this case, at the pharmaceutical outpatient clinic of the department of cardiovascular internal medicine, the pharmacist confirmed that the patient did not have issues with medication adherence. Further, there were no additional prescriptions or over-the-counter medications and no significant changes in the patient’s hepatic or renal function. On one hand, the pharmacist suggested to the physician at the outpatient clinic that the decrease in vitamin K intake due to anorexia and the change from bosentan to macitentan resulted in the cancellation of CYP2C9 and CYP3A4 enzyme induction by bosentan, enhancing the anticoagulant effect of warfarin, and the patient’s PT-INR could be prolonged.

Warfarin is metabolized by multiple drug-metabolizing enzymes, including CYP2C9, CYP3A4, and CYP1A2 [4]. Prior to changing pulmonary hypertension medications from bosentan to macitentan, bosentan could have increased the hepatic metabolism of warfarin by inducing CYP2C9 and CYP3A4 activity and decreasing its anticoagulant effects. There are several reports on the decreased effect of the concomitant administration of warfarin and bosentan. For example, a previous study has shown a 63.6% increase in the warfarin maintenance dose from 27.5 to 45 mg/week after the introduction of bosentan [5]. Moreover, another research revealed a 38% decrease in the area under the plasma concentration time curve of warfarin, together with 23% to 38% decrease in the PT in the bosentan group compared with the placebo group [6].

Conversely, the drug–drug interactions of warfarin and macitentan are not described in the Japanese package inserts of both drugs and UpToDate Lexidrug (Wolters Kluwer) [7]. The pharmacokinetics of combined macitentan and warfarin in adult male Caucasian patients did not change compared with that of either drug alone, and there were no drug–drug interactions between macitentan and warfarin [8]. Therefore, the change from bosentan to macitentan cancelled the attenuation of anticoagulant effect of warfarin via drug–drug interaction with bosentan, and the continuation of warfarin therapy at the same dose resulted in a hyper-prolonged PT-INR.

Warfarin is a coumarin derivative with a vitamin K-like structure, and it exerts its anticoagulant effect by inhibiting the production of vitamin K-dependent blood clotting factors in the liver [9]. Vitamin K intake at a dose of 150 μg/day is considered appropriate for patients taking warfarin [10]. Vitamin K intake at a dose of >150 μg/day may affect the anticoagulant effect of warfarin. Therefore, it is essential to maintain the daily intake of vitamin K [11]. In this case, the increased anticoagulant effect of warfarin caused by the decreased vitamin K intake, which is attributed to anorexia, might have contributed to the increased PT-INR. In fact, a previous study showed a case of rapid PT-INR prolongation (>12) with decreased appetite caused by urinary tract infection [12].

The serum albumin (Alb) concentration was also more likely to decrease on the day of admission (3.9 g/dL) probably due to decreased appetite. The half-life of Alb was as long as 14–21 days [13]. Thus, the lowest value (3.1 g/dL) was observed on day 5 of admission. Warfarin highly binds to serum Alb. Hence, a decrease in Alb concentrations temporarily increases the free warfarin concentration [14]. Thus, its anticoagulant effects may be stronger when the Alb concentration is low. Subsequently, the Alb concentrations started to increase with improvement in appetite during hospitalization.

In this case, the patient had a low Hb level and was anemic at the time of the outpatient visit. In a double-blind, placebo-controlled, randomized study on macitentan (the SERAPHIN study), the 10 mg/day macitentan group was more likely to present with anemia (13.2%) than the placebo group (3.2%). However, the incidence of anemia leading to treatment discontinuation was similar in both groups [15]. After informing and discussing with the physician that anemia was reported as an adverse effect of macitentan in the package insert, and the cause of anemia progression could be drug-related adverse effect. Therefore, the physician decided to resume bosentan instead of macitentan. During admission, the macitentan treatment was discontinued, and transfusion therapy resulted in an increase in Hb levels and improvement in anemia symptoms. When resuming bosentan treatment, the physician planned to prescribe warfarin at the current dose of 2 mg/day. However, the pharmacist considered that bosentan would decrease the anticoagulant effect of warfarin and lower PT-INR. Hence, the pharmacist proposed to increase the warfarin dose to 3.75 mg/day, which is the same dose with concomitant administration with bosentan before. Consequently, the PT-INR remained within the therapeutic range.

Pharmacist intervention in the outpatient clinic of cardiovascular internal medicine was effective in reducing the risk of bleeding in patients receiving antithrombic therapy [2]. Although it was important for pharmacists to conduct sufficient interviews and careful monitoring after drug changes in collaboration with physicians in the outpatient setting, the pharmaceutical outpatient clinic was offered to specific physicians only because of the lack of workforce in our hospital. If pharmacist outpatient clinics were offered to all patients taking antithrombotic drugs, the pharmacist could propose appropriate warfarin dosage, as in this case, when the prescription is changed from bosentan to macitentan. Therefore, in the future, pharmaceutical outpatient clinics should be expanded to provide appropriate pharmacotherapy management.

Based on the patient’s complaint (anorexia) and drug history at the pharmacist outpatient clinic, the pharmacist suggested that PT-INR prolongation was caused by the enhanced effect of warfarin via drug–drug interaction. Moreover, as the pharmacist collaborated with the physician after hospitalization, the patient’s PT-INR was managed within the therapeutic range, and the patient was successfully discharged from the hospital. We believe that active pharmacist intervention in the patient’s pharmaceutical treatment from the outpatient clinic through hospitalization and discharge can improve the patient’s condition and prevent the recurrence of adverse drug effects.
